# On the Difficulty of Budget Allocation in Claims Problems with Indivisible Items and Prices

**DOI:** 10.1007/s10726-021-09750-1

**Published:** 2021-06-29

**Authors:** Teresa Estañ, Natividad Llorca, Ricardo Martínez, Joaquín Sánchez-Soriano

**Affiliations:** 1 Centro de Investigación Operativa (CIO), Universidad Miguel Hernández de Elche, Elche, Spain; 2 Departmento de Teoría e Historia Económica, Universidad de Granada, Granada, Spain

**Keywords:** Claims problems, Indivisible items, Equal treatment of equals, Non-wastefulness, Manipulability

## Abstract

In this paper we study the class of claims problems where the amount to be divided is perfectly divisible and claims are made on indivisible units of several items. Each item has a price, and the available amount falls short to be able to cover all the claims at the given prices. We propose several properties that may be of interest in this particular framework. These properties represent the common principles of fairness, efficiency, and non-manipulability by merging or splitting. Efficiency is our focal principle, which is formalized by means of two axioms: *non-wastefulness* and *Pareto efficiency*. We show that some combinations of the properties we consider are compatible, others are not.

## Introduction

Resource allocation problems have been extensively studied in the literature for their relevance, particularly when there is a shortage of resources. Decisions made about the allocation problem can lead to grievances and tough negotiations. For this reason, it is interesting to know what difficulties are faced by those who have to make decisions about the allocation of resources, particularly when they are scarce. Claims problems or bankruptcy problems are one type of these resource allocation problems. In the classical claims problem, introduced by O’Neill (1982), a central authority has to decide how to divide among the creditors the liquidation value of a firm that goes into bankruptcy. Obviously, this liquidation value does not exceed the debt to the creditors.^1^ Usually, both the creditors’ claims and the value to be divided are perfectly divisible. In this work we present a novel model where the amount to be divided is in money (and therefore divisible) but the demands are made on indivisible items,^2^ each with an associated price. Imagine a health authority responsible for several hospitals that, as in the case of the COVID-19 pandemic, has to allocate to all of them the scarce resources. Each of those hospitals will ask for several medical items (beds, ventilators, defibrillators...). Each of those items has a market price and the health authority has a budget with which to buy them. How many items of each type should be allocated to each hospital taking into consideration their claims, the prices and the available budget? The fact that the demands are expressed in indivisible units of different items, while the amount to divide is continuous, presents several decision difficulties that we analyze in this work.

To be more precise, in our setting, a claims problem can be condensed into five elements. Namely: a set of *agents* of the claimants, a set of possible *items* demanded, a vector of *prices* of those items, a matrix of *claims* that specifies the number of units each agent claims on each item, and the available amount (called *estate*). In addition, it happens that the estate falls short to be able to cover the whole claim at the given prices. A rule is a way in which to solve claims problems. In particular, we consider multi-valued functions, which may be more convenient in order to deal with the indivisibilities in the model.

There are other authors that have studied allocations problems with indivisibilities. In some cases both the budget and the demands are integers (Chen 2015; Herrero and Martínez 2011, 2008a, 2008b), while in other papers the estate is indivisible but the claims are continuous (Fragnelli et al. 2016, 2014). With respect to those works, the novelty we present is twofold. One, the claims are on multiple items. And two, and more significant, the existence of prices, which allow us to consider and combine a continuous estate with indivisible claims.

Following the axiomatic methodology, we wonder if rules exist that satisfy suitable combinations of properties (called *axioms*) that represent criteria on efficiency, fairness, and stability. A claims problem represents a situation where there is a shortage, and therefore the first requirement that comes to mind is to use the limited budget efficiently, trying to satisfy most of the claims with the least amount of money. We implement this principle by means of the *non-wastefulness* condition, which simply says that we would waste as little estate as possible.^3^ As an alternative to non-wastefulness, we also analyze the *Pareto efficiency* condition. While the former represents efficiency from the point of view of a central authority/government (wasting the least of the budget), the latter takes the perspective of the agents (their allocation cannot be improved at the cost of worsening other individual). We also study other properties that implement several principles of fairness and stability. For the former we consider *weak equal treatment of equals* (agents with equal claims should get equal allotments), while for the latter we impose *non-manipulability by merging or splitting* (agents cannot manipulate their assignments either by splitting or merging their claims).

Interestingly, the finding of non-wasteful rules is closely related with a well-known programming problem, the so-called bounded knapsack problem.^4^ Since the seminal paper by Dantzig (1957), several extensions have been widely studied due to their practical applications Kellerer et al. (2010), including choice theory Feuerman and Weiss (1973). As examples of interest which relate to our situation, Darmann and Klamler (2014) study how to share the estate in a continuous setting by means of optimal solutions, and Arribillaga and Bergantiños (2019) analyze two rules related to the Shapley value of an optimistic game.

In the context of claims problems with indivisibilities, several papers have proposed different type of solutions. Moulin (2000), Herrero and Martínez (2008a), Chen (2015) use priority methods, while Giménez-Gómez and Vilella Bach (2012) adopt a P-rights recursive process, described in Giménez-Gómez and Marco-Gil (2014), to ensure weak order preservation.^5^ Discrete claim models have been widely used to deal with scarce resources in technological problems such as mobile radio networks (Lucas-Estañ et al. 2012; Gozálvez et al. 2012) or social problems such as apportionment problems Sánchez-Soriano et al. (2016). On the other hand, in claims problems with multi-dimensional and perfectly divisible claims Calleja et al. (2005) introduce the run to the bank rule, while Bergantiños et al. (2011) present several characterizations of the constrained equal awards rule, and Moreno-Ternero (2009) studies the proportional rule.

With respect to our findings, we show that if we require non-wastefulness together with properties such as weak equal treatment of equals Young (1988; 1994) and non-manipulability (O’Neill 1982; de Frutos 1999; Ju et al. 2007), exemption Herrero and Villar (2001), conditional full compensation (Herrero and Villar 2002; Herrero and Martínez 2008b; securement (Moreno-Ternero and Villar 2004), or self-duality Aumann and Maschler (1985), we end up with an impossibility. As for Pareto efficiency, it is also incompatible with weak equal treatment of equal and non-manipulability. However, this notion of efficiency is compatible with exemption, conditional full compensation, and weak securement.

The rest of the paper is structured as follows: In Sect. 2 we set the model. In Sect. 3 we present the three core properties we analyze, including non-wastefulness. In Sect. 4 we explore the compatibility of the axioms. In Sect. 5 we study several protective and duality properties, and we illustrate their incompatibilities with non-wastefulness. In Sect. 6 we analyze an alternative formulation of the efficiency principle: Pareto efficiency. Finally, Sect. 7 concludes with a final discussion.

## The mathematical Model

Let $N=\{1,\ldots ,n\}$ be the set of agents and let $H=\{1,\ldots , h\}$ be the set of possible items, whose prices are given by $p=(p_1,\ldots ,p_h) \in {\mathbb {R}}^h_+$.

A claims problem with indivisible items and different prices, or simply a **problem**, represents a situation in which a perfectly divisible quantity, $E\in {\mathbb {R}}_{++}$ (called **estate**) must be distributed among agents in *N* according to their demands. Those demands are described by means of a matrix of claims $c \in {\mathbb {Z}}_+$ that has as many rows as agents, and as many columns as items

$$\begin{aligned} c= \left( \begin{array}{cccc} c_{11} &{} c_{12} &{} \ldots &{} c_{1h} \\ c_{21} &{} c_{22} &{} \ldots &{} c_{2h} \\ \vdots &{} \vdots &{} \ddots &{} \vdots \\ c_{n1} &{} c_{n2} &{} \ldots &{} c_{nh} \\ \end{array} \right) , \end{aligned}$$

where $c_{ig} \in {\mathbb {Z}}_+$ indicates the amount of item *g* claimed by agent *i*. In any claims problem, the estate falls short to fully cover all the demands, that is, $\sum _{i=1}^n \sum _{g=1}^h c_{ig} p_g \ge E$.

Therefore, a problem is given by a tuple $a=(N,H,p,c,E)$, where *N* is the set of agents, *H* is the set of items, *p* is the vector of prices, *c* is the matrix of claims, and *E* is the estate. Since the elements *N*, *H*, and *p* are fixed throughout the paper, when no confusion arises we simply write the claims problem as $a=(c,E)$. Let ${\mathbb {A}}$ be the set of all problems:

$$\begin{aligned} {\mathbb {A}}=\left\{ a=(c,E) \in {\mathbb {Z}}^{n \times h}_{+} \times {\mathbb {R}}_{++} : \Vert c \cdot p \Vert = \sum _{i=1}^n \sum _{g=1}^h c_{ig} p_g \ge E \right\} . \end{aligned}$$

An **allocation** for $a \in {\mathbb {A}}$ is a distribution of the estate among the agents that specifies how many items of each price are awarded to each agent. Thus, it is a matrix $x \in {\mathbb {Z}}^{n \times h}_{+}$ that satisfies the following two conditions:

- Each agent receives a non-negative amount of each type of item, which is not larger than her claim:
  $$\begin{aligned} 0 \le x_{ig} \le c_{ig} \quad \text { for all } i\in N \text { and all } g \in H. \end{aligned}$$
- The overall cost does not exceed the available estate:
  $$\begin{aligned} \Vert x \cdot p\Vert =\sum _{i=1}^n \sum _{g=1}^h x_{ig} p_g \le E. \end{aligned}$$

We denote by *X*(*a*) the set of all feasible allocations for the problem $a \in {\mathbb {A}}$.

All standard models on claims problems impose that the estate must be exhausted and nothing remains without being allocated. Notice that, Condition (b) relaxes this requirement and part of the estate may be unassigned. Otherwise, if equality is imposed, the set of allocations will be empty for some problems.

### Example 1

Let $N=\{1,2,3,4\}$, $H=\{1,2,3\}$, and $p=(2,4,5)$. For the problem $a=(N,H,p,c,E)$, where $E=16$ and

$$\begin{aligned} c= \left( \begin{array}{ccc} 1 &{} 0 &{} 2 \\ 0 &{} 0 &{} 1 \\ 0 &{} 0 &{} 1 \\ 1 &{} 1 &{} 2 \\ \end{array} \right) . \end{aligned}$$

The overall claimed amount is^6^

$$\begin{aligned} \Vert c \cdot p\Vert = \sum _{i=1}^4 \sum _{g=1}^3 c_{ig} p_g = 38 \ge 16 = E. \end{aligned}$$

Three possible allocations for this problem are

$$\begin{aligned} x= \left( \begin{array}{ccc} 0 &{} 0 &{} 0 \\ 0 &{} 0 &{} 0 \\ 0 &{} 0 &{} 0 \\ 0 &{} 0 &{} 0 \\ \end{array} \right) , \quad x'= \left( \begin{array}{ccc} 0 &{} 0 &{} 1 \\ 0 &{} 0 &{} 1 \\ 0 &{} 0 &{} 0 \\ 0 &{} 0 &{} 1 \\ \end{array} \right) , \quad x''= \left( \begin{array}{ccc} 0 &{} 0 &{} 1 \\ 0 &{} 0 &{} 0 \\ 0 &{} 0 &{} 0 \\ 1 &{} 1 &{} 1 \\ \end{array} \right) . \end{aligned}$$

All the three previous matrices satisfy the two conditions to be allocations of the problem, but they differ in that part of the estate that is wasted or non-used $(E-\Vert x \cdot p\Vert )$. Hence, for the null allocation *x* we have that $\Vert x \cdot p\Vert =0$, so all the estate is left, for $x'$, $\Vert x' \cdot p\Vert = 15$, so only one unit is left, while in $x''$, $\Vert x'' \cdot p\Vert = 16$, hence the estate is exhausted.

A **rule** is a way of selecting allocations. In our setting, it is a correspondence, 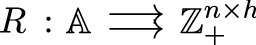, that selects, for each problem $a \in {\mathbb {A}}$, a non-empty subset of allocations $R(a) \subseteq X(a)$.

We next present some examples of rules than can be used to solve a claims problem with indivisible items and prices.

The first rule is straightforward, it simply stipulates that no agent receives anything. Obviously, from a practical perspective this rule is pointless, but it is useful from a theoretical point of view, since it can be used to illustrate certain problems with the properties of the rules.

**Null rule**, $R^N$. For each $a \in {\mathbb {A}}$ and each $x \in X(a)$,

$$\begin{aligned} x \in R^N(a) \; \Leftrightarrow \; x_{ig}=0 \; \forall i \in N \text { and } \forall g \in H \end{aligned}$$

A rule is a multi-valued mapping, so it may select more than one allocation. The next proposal is an extreme case, since it selects the whole set of allocations *X*(*a*). It is the counterpart of the null rule.

**Greedy rule**, $R^G$. For each $a \in {\mathbb {A}}$,

$$\begin{aligned} R^G(a)=X(a). \end{aligned}$$

Let $\succ _N$ be an ordering on the set of claimants *N*, where $i \succ _N j$ means *i* has priority over *j*. Let $\succ _H$ be an ordering on the set of items *H*, where $f \succ _H g$ means *f* has priority over *g*. Consider now a rule as the following *modus operandi*: the agents arrive one at a time in the ordering $\succ _N$, and try to fully satisfy them, starting with the items with the highest priority in $\succ _H$. This process continues until, eventually, the estate runs out. The formal definition of this rule is given below.

**Agent-item priority arrival rule**, $R^{AIPA}$. For each $a \in {\mathbb {A}}$ and each $x \in X(a)$,

$$\begin{aligned} x \in R^{AIPA}(a) \; \Leftrightarrow \; \left[ x_{ig}>0 \Rightarrow x_{if}=c_{if} \, \forall f \succ _H g \text { and } x_{jf}=c_{jf} \, \forall j \succ _N i \; \forall f \in H \right] . \end{aligned}$$

As a result of the application of this rule, agents with higher priority are satisfied before those with lower priority. Besides, for each agent, more relevant items are fully served first. Consider, for example, the impact of the COVID-19 pandemic in a country whose regions (agents) are significantly heterogenous with respect to the pressure levels of their ICUs. It is natural to prioritize those regions with more pressing needs. In addition, some items (ventilators, for instance) are more critical than others for the patients survival. The agent-item priority arrival rule could be appropriate for such a kind of situations.

Several generalizations of the previous rule can be defined by, for example, considering different orderings of items for different claimants. Alternatively, instead of applying $\succ _N$ and then $\succ _H$, it is also possible to do the converse.

Another rule based on priority which better captures the idea behind rules with similar name in other settings (see Thomson 2019) is the following. Given an ordering $\succ _N$ on the set of claimants, agents arrive one at a time in the ordering. The first agent in the ordering selects the set of items so that she maximizes the value of her choice subject to the budget constrained given by *E*. Let $E^1$ be the remaining estate. Now, the second agent in the ordering selects the set of items so that she maximizes the value of her choice subject to the budget constrained given by $E^1$. We continue the process until the estate, eventually, runs out.

**Agent priority arrival rule**, $R^{APA}$. For each $a \in {\mathbb {A}}$ and each $x \in X(a)$,

$$\begin{aligned} x \in R^{APA}(a) \; \Leftrightarrow \; \left[ x_{jg}>0 \Rightarrow x_{ig}=c_{ig} \, \forall i \succ _N j \right] . \end{aligned}$$

Notice that, in comparison with the agent-item priority arrival rule, this rule consumes more budget in each step of the process, since there is no ordering on the items that restricts the agent’s choice. Therefore, these two rules are suitable for similar situations, depending on whether some item must or must no be prioritized.

The next rule is a two-step process. First, the estate is equally divided among the items ($\frac{E}{h}$ for each one). And second, for each item, amounts as equal as possible are assigned to all claimants subject to no-one receiving more than her claim.^7^ This rule can be used when the central planner is interested in allocating the budget as equally as possible not only among the agents but also among the different items.

**Equal-by-item rule**, $R^{EI}$. For each $a \in {\mathbb {A}}$ and each $x \in X(a)$,

$$\begin{aligned} x \in R^{EI}(a) \; \Leftrightarrow \; {\left\{ \begin{array}{ll} |x_{ig}-x_{jg}| \le 1 \text { for all } i,j \in N \\ p_g \left( \sum _{i=1}^n x_{ig} \right) \le \frac{E}{h} \\ p_g \left( 1 + \sum _{i=1}^n x_{ig} \right) > \frac{E}{h}. \end{array}\right. } \end{aligned}$$

In the previous definition, for each possible item, the first condition states that the difference between the awards of two agents is no larger than 1. The second condition imposes that the overall cost of all assigned units does not exceed the share of the estate for this item. Finally, the third one says that the part of the estate corresponding to this item is efficiently distributed, wasting as little as possible.

Several variations of the previous rule are possible. For example, we can consider the distribution of the estate among the items different from the uniform split. We can also restrict the set of allocations by introducing an ordering on *N* as a tie breaking scheme. Besides, we can obviate the third condition on the efficient usage of the estate.

Another interesting rule could be obtained by applying the two step process of the equal by item rule but to agents. Namely, some kind of equal by agent rule. For each agent we select a set of items whose price is smaller or equal than $\frac{E}{n}$ and such that adding a new item the price is larger than $\frac{E}{n}$. Later the remaining budget is assigned to any set of agents spending as much as possible.

**Equal-by-agent rule**, $R^{EA}$. For each $a \in {\mathbb {A}}$ and each $x \in X(a)$,

$$\begin{aligned} x =z+y \in R^{EA}(a) \; \Leftrightarrow \; {\left\{ \begin{array}{ll} z,y \in {\mathbb {Z}}^{n\times h}_+\\ \sum _{g=1}^{h}p_gz_{ig}\le \frac{E}{n}, \forall i\in N \\ \sum _{g=1}^{h}p_gz'_{ig}> \min \{\sum _{g=1}^{h}p_gc_{ig},\frac{E}{n}\}, \forall z' > z, \forall i\in N \\ \sum _{i=1}^{n}\sum _{g=1}^{h} p_gy_{ig} \le E - \Vert z \cdot p\Vert \\ \sum _{i=1}^{n}\sum _{g=1}^{h} p_gy_{ig} \ge \sum _{i=1}^{n}\sum _{g=1}^{h} p_gy'_{ig}, \forall y' \in X(a'), \end{array}\right. } \end{aligned}$$

where $z' > z$ means that there is at least one cell *ig* such that $z'_{ig} > z_{ig}$, and the others are greater or equal; and $a'=(N,H,p,c-z,E- \Vert x \cdot p\Vert )$.

### Example 2

Continuing with the claims problem of Example 1, these are the allocations selected by each of the rules described above.

- Null rule.
  $$\begin{aligned} R^N(a)= \left\{ \left( \begin{array}{ccc} 0 &{} 0 &{} 0 \\ 0 &{} 0 &{} 0 \\ 0 &{} 0 &{} 0 \\ 0 &{} 0 &{} 0 \\ \end{array} \right) \right\} . \end{aligned}$$
- Greedy rule.
  $$\begin{aligned} R^G(a)=X(a). \end{aligned}$$
- Agent-item priority arrival rule. Let us suppose that the ordering $\succ _N$ and $\succ _H$ are such that $1 \succ _N 2 \succ _N 3 \succ _N 4$ and $3 \succ _H 2 \succ _H 1$. Given $\succ _N$, we start with Agent 1. When this agent is fully honored, the remaining estate is $16-(2 \cdot 5 + 1 \cdot 2) = 4$. Claimant 2 is the next in line. According to $\succ _H$ we should start by awarding her demand on item 3. However, the unit cost of item 3 she is claiming is 5, which exceeds the available estate. Then, the process stops and the allocation is the following:
  $$\begin{aligned} R^\text {AIPA}(a)= \left\{ \left( \begin{array}{ccc} 1 &{} 0 &{} 2 \\ 0 &{} 0 &{} 0 \\ 0 &{} 0 &{} 0 \\ 0 &{} 0 &{} 0 \\ \end{array} \right) \right\} . \end{aligned}$$
- Agent priority arrival rule. Let us suppose again that the ordering $\succ _N$ is such that $1 \succ _N 2 \succ _N 3 \succ _N 4$. We start with Agent 1. He is fully honored obtaining $x_{11}=1$ and $x_{13}=2$, and the remaining budget is 4. Agents 2 and 3 cannot obtain anything since the items they demand have a price of 5. Finally, Agent 4 can choose 1 unit of item 1 or 1 unit of item 2, but if we consider agents are maximizers of the budget allocated to them, then the only alternative is $x_{42}=1$. Then, the process ends and the rule gives the following allocation:
  $$\begin{aligned} R^\text {APA}(a)= \left\{ \left( \begin{array}{ccc} 1 &{} 0 &{} 2 \\ 0 &{} 0 &{} 0 \\ 0 &{} 0 &{} 0 \\ 0 &{} 1 &{} 0 \\ \end{array} \right) \right\} . \end{aligned}$$
  Note that if $c_{41}$ had been 2 instead of 1, then $R^\text {APA}(a)$ would have two possible allocations.
- Equal-by-item rule. First, we equally divide the estate among the items ($E_1=E_2=E_3=\frac{16}{3}$). We start with item 1. Since $p_1=2$, $E_1$ is enough to fully cover the demands of Agents 1 and 4. The same argument applies to item 2. Finally, item 3 must be rationed because the cost of honoring all the demands exceeds the share of the estate devoted to this item. With $E_3=\frac{16}{3}$ we can only distribute 1 unit at a price $p_3=5$. Following the definition of the rule, this unit may be assigned to any agent. Therefore, the equal-by-item rule selects the following allocations:
  $$\begin{aligned} R^\text {EI}(a)= \left\{ \left( \begin{array}{ccc} 1 &{} 0 &{} 1 \\ 0 &{} 0 &{} 0 \\ 0 &{} 0 &{} 0 \\ 1 &{} 1 &{} 0 \\ \end{array} \right) , \left( \begin{array}{ccc} 1 &{} 0 &{} 0 \\ 0 &{} 0 &{} 1 \\ 0 &{} 0 &{} 0 \\ 1 &{} 1 &{} 0 \\ \end{array} \right) , \left( \begin{array}{ccc} 1 &{} 0 &{} 0 \\ 0 &{} 0 &{} 0 \\ 0 &{} 0 &{} 1 \\ 1 &{} 1 &{} 0 \\ \end{array} \right) , \left( \begin{array}{ccc} 1 &{} 0 &{} 0 \\ 0 &{} 0 &{} 0 \\ 0 &{} 0 &{} 0 \\ 1 &{} 1 &{} 1 \\ \end{array} \right) \right\} . \end{aligned}$$
- Equal-by-agent rule. First, we equally divide the estate among the agents, $\frac{16}{4}=4$. With this distribution of the budget there are only two alternatives
  $$\begin{aligned} \left\{ \left( \begin{array}{ccc} 1 &{} 0 &{} 0 \\ 0 &{} 0 &{} 0 \\ 0 &{} 0 &{} 0 \\ 1 &{} 0 &{} 0 \\ \end{array} \right) , \left( \begin{array}{ccc} 1 &{} 0 &{} 0 \\ 0 &{} 0 &{} 0 \\ 0 &{} 0 &{} 0 \\ 0 &{} 1 &{} 0 \\ \end{array} \right) \right\} . \end{aligned}$$
  In the first case 12 units of the budget are left and in the second case 10 units are left. In both cases, the allocations spending as much as possible of the remaining budget are
  $$\begin{aligned} Y= \left\{ y \in {\mathbb {Z}}^{4 \times 3}_{+}:\sum _{i=1}^{4}y_{i3}=2,y_{i1}=y_{i2}=0, \forall i \in N \right\} . \end{aligned}$$
  Therefore,
  $$\begin{aligned} R^\text {EA}(a)=\left\{ \left( \begin{array}{ccc} 1 &{} 0 &{} 0 \\ 0 &{} 0 &{} 0 \\ 0 &{} 0 &{} 0 \\ 1 &{} 0 &{} 0 \\ \end{array} \right) +y, \left( \begin{array}{ccc} 1 &{} 0 &{} 0 \\ 0 &{} 0 &{} 0 \\ 0 &{} 0 &{} 0 \\ 0 &{} 1 &{} 0 \\ \end{array} \right) +y : y \in Y \right\} \end{aligned}$$

All the previous rules (and their possible generalizations) arise as natural ways to solve a claims problem with indivisible items of different prices. Some of them may be more appealing than others. There are rules (like the null rule) that make very inefficient usage of the available estate, which is quite undesirable in a situation of shortage. Other proposals may result in very ,,unfair" allocations. Thus, the priority arrival rules, for instance, do not take into account any minimal principle of justice. Some claimants are fully satisfied while others get nothing. Besides, solutions in the spirit of the equal-by-item or the equal-by-agent rules are easily manipulated by the agents merging or splitting their claims. In the next sections we deliberate over the existence of rules with good properties that can be applied to our claims problems.

## Three Core Properties

In this section we present three minimal requirements a rule should satisfy, which are quite standard in the literature on claims problems. The restrictions they impose are so slight that they are usually compatible (O’Neill 1982; de Frutos 1999; Ju et al. 2007; Estañ et al. 2020). The first property stipulates that in a rationing situation we should waste as little as possible. The second property is a minimal criterion on fairness, and states that agents with equal claims should be equally treated. Finally, the last property makes the rule immune to certain manipulations by the agents. To summarize, efficiency, fairness, and non-manipulability will be the core requirements we impose as starting point.

As we have seen in Example 2, it may happen that not all the allocations completely exhaust the estate. Given the nature of the problem, it is natural to require that the rule chooses an allocation that misuses the estate as little as possible. This is the counterpart of the *efficiency* requirement in claims problems with continuous claims and estate, which states that the entire amount available should be allocated (see, Thomson (2003) and Thomson (2015), for example). 

**Non-wastefulness** For each $a \in {\mathbb {A}}$, if $x \in R(a)$, then there is no other allocation $x' \in X(a)$ such that $E - \Vert x' \cdot p\Vert < E - \Vert x \cdot p\Vert$.

The next property introduces a minimal condition of equality, imposing that individuals with the same claims should be treated equally. Obviously, because of the nature of the problem and the existence of indivisible items, complete equality is difficult to achieve (if not impossible in most cases). So, we modify the condition to require that agents with equal claims must obtain allocations as equal as the indivisibility allows, and equal agents must have the same opportunities. That is, if two individuals demand the same units of all items, then (i) their allocations differ, at most, by one unit per item (in each allocation their awards are as equal as the indivisibility permits), and (ii) the set of selected allocations is symmetric with respect to these two agents (both have the same opportunities to receive one unit more than the other).

**Weak equal treatment of equals** For each $a \in {\mathbb {A}}$ and each $\{i,j\} \subseteq N$, if $c_{ig}=c_{jg} \forall g \in H$, then for all $x \in R(a)$ it holds that

- for all $g \in H$, $|x_{ig} - x_{jg}| \le 1$, and
- for each $g \in H$, there is $x' \in R(a)$, such that $x'_{ig}=x_{jg}$, $x'_{jg}=x_{ig}$ and the rest of cells of $x'$ are the same as in *x*.

Finally, the next principle says that the rule is immune to manipulation. More precisely, it states that agents cannot manipulate the allocation by either merging or splitting their demands. If a group of individuals merge into a single claimant whose demand (for each item) is the sum of the demands of all the members of such a group, then the allocation of this phantom claimant should coincide with the aggregate allocation the group would have obtained if they had concurred separately. Dually, if an agent splits into a group of different individuals, the aggregate allocation should coincide with the allotment this single agent would have received.^8^

Before formalizing the axiom, we must point out that we are working with correspondences, which means that the outcome of a rule is a set of allocations. Therefore, comparing two outcomes requires comparing two sets, and several possibilities arise: $S=T$, $S \subseteq T$, or $T \subseteq S$. From those alternatives, *non-manipulability by merging or splitting* states that (1) for each allocation in the shrunk problem (the problem with the phantom agent) there must exist a corresponding allocation in the expanded problem (without the phantom agent), and (2), for each allocation in the expanded problem there must exist a corresponding allocation in the shrunk problem. 

**Non-manipulability by merging or splitting** For each $(N,c,E), (N',c',E) \in {\mathbb {A}}$ with $N' \subset N$, if there is $i \in N'$ such that the following two conditions hold

1. $$c'_{ig} = c_{ig} + \sum _{j \in N \backslash N'} c_{jg}$$ for all $$g \in H$$
2. $c'_{jg}=c_{jg}$ for all $j \in N' \backslash \{i\}$ and for all $g \in H$,

then

- $\forall x' \in R(N',c',E)$ there exists $x \in R(N,c,E)$ such that $x'_{ig} = x_{ig} + \sum _{j \in N \backslash N'} x_{jg}$ $\forall g \in H.$
- $\forall x \in R(N,c,E)$ there exists $x' \in R(N',c',E)$ such that $x'_{ig} = x_{ig} + \sum _{j \in N \backslash N'} x_{jg}$ $\forall g \in H.$

Note that the definition of non-manipulability by merging or splitting states that any allocation that a group of agents could receive through their merger could also have been obtained by remaining separate, and conversely, any allocation that a group of agents could receive by remaining separate could also have obtained through their merger.

## Compatibility Results

In the previous section, we have formalized three basic properties that implement principles of efficiency, fairness, and non-manipulability. Now, we explore their compatibility, that is, we analyze if rules exist that satisfy all or some of those properties.

Given a problem $a \in {\mathbb {A}}$. Consider the following integer linear programming problem (ILP, for short):

$$\begin{aligned} \left. \begin{aligned} \min _{x \in {\mathbb {Z}}^{n \times h}_+}&E-\sum _{i=1}^n \sum _{g=1}^h p_g x_{ig} \\ \text {s.t.: }&\sum _{i=1}^n \sum _{g=1}^h p_g x_{ig} \le E \\&0 \le x_{ig} \le c_{ig}, \; \forall i \in N, \forall g \in H \end{aligned} \right\} \end{aligned}$$

or equivalently,

1 $$\begin{aligned} \left. \begin{aligned} \max _{x \in {\mathbb {Z}}^{n \times h}_+}&\sum _{i=1}^n \sum _{g=1}^h p_g x_{ig} \\ \text {s.t.: }&\sum _{i=1}^n \sum _{g=1}^h p_g x_{ig} \le E \\&0 \le x_{ig} \le c_{ig}, \; \forall i \in N, \forall g \in H \end{aligned} \right\} \end{aligned}$$

Let us denote by *ILP*(*a*) the set of all optimal solutions for the program in (1). It is easy to observe that a rule *R* satisfies non-wastefulness if it is a selection of solutions of the previous optimization problem, i.e., $R(a) \subseteq ILP(a)$ for all $a \in {\mathbb {A}}$. The integer linear program defined by (1) belongs to the class of bounded knapsack problems.^9^ Since the seminal paper by Dantzig (1957) several extensions have been widely studied due to their practical applications ( Kellerer et al. 2010). In general, the solutions of a bounded knapsack problem cannot be obtained in polynomial time. Besides, most of the algorithms are heuristic, and they are usually unable to find all the possible allocations. In other words, finding the set of non-wasteful allocations of the claims problem (and therefore the goal of efficiency) is a very hard task, if not impossible. However, despite these technical difficulties, the following results shows that some interesting conclusions can be derived.

### Theorem 1

*There are rules that satisfy non-wastefulness, weak equal treatment of equals and non-manipulability by merging or splitting if and only if there are rules that satisfy those properties for the subclass of problems with* $|H|=1$.

### Proof

It is obvious that if there are rules that satisfy non-wastefulness, weak equal treatment of equals and non-manipulability by merging or splitting then those rules satisfy those properties for the subclass of problems with $|H|=1$.

Conversely, let us suppose that there are rules that satisfy non-wastefulness, weak equal treatment of equals and non-manipulability by merging or splitting for the subclass of problems with $|H|=1$. Let *R* be one of those rules, then we define the following procedure for all problems $a=(N,H,p,c,E) \in {\mathbb {A}}$:

First, we consider the bankruptcy problem $b(a)=({\mathcal {N}},d,u,E)$ given by

- The set of claimants ${\mathcal {N}} = H$, i.e the claimants are the different types of items.
- $d_g = p_g \sum _{i \in N} c_{ig}$, for all $g \in H$, i.e. the claim of item *g* is exactly the total amount claimed by all agents for the item *g*.
- *u* is a vector of utility functions defined for each $g \in {\mathcal {N}} = H$ as follows:
  $$\begin{aligned} u_g(y) = \left\lfloor \frac{y}{p_g} \right\rfloor \times p_g, \end{aligned}$$
  where $\lfloor r \rfloor$ is the integer part of $r \in {\mathbb {R}}$.
- Finally, the estate *E* is exactly the same as in $a=(N,H,p,c,E) \in {\mathbb {A}}$.

This bankruptcy problem is closely related to those studied in Gozálvez et al. (2012), Lucas-Estañ et al. (2012) and Carpente et al. (2013), in which the agents are considered to have different utility functions in the sense that the same part of the estate has different degrees of satisfaction for the claimants. In our particular case, for example, Claimant 1 must receive at least $p_1$ units of the estate to obtain one level of satisfaction, i.e. one item of type $1 \in H$, and this can be different for each claimant $g \in {\mathcal {N}} = H$.

Second, we distribute the estate *E* amongst the claimants ${\mathcal {N}} = H$ by solving the following linear program:

2 $$\begin{aligned} \left. \begin{aligned} \max _{y \in {\mathbb {R}}^{h}_+}&\sum _{g=1}^h u_g(x_g) \\ \text {s.t.: }&\sum _{g=1}^h y_{g} \le E \\&0 \le y_{g} \le p_g \sum _{i=1}^n c_{ig}, \; \forall g \in {\mathcal {N}} = H \end{aligned} \right\} \end{aligned}$$

Note that every optimal solution of the integer linear program defined by (1) results in a feasible solution of the linear program defined by (2), and this must be optimal because, otherwise, we would be able to find a better solution for the problem given in (1) from an optimal solution of the problem given in (2). We denote by *LP*(*b*(*a*)) the set of all optimal solutions ${\bar{x}}$ of the linear program given by (2) such that $\frac{{\bar{y}}_g}{p_g} \in {\mathbb {Z}}_{+}, \forall g \in {\mathcal {N}}$.

Third, for every optimal solution ${\bar{y}} \in LP(b(a))$, we consider the family of problems ${\bar{a}}_g=(N,H=\{g\}, p_g, c_g, {\bar{y}}_g)$, where $c_g=(c_{1g},\ldots ,c_{ng})$.

Finally, for each problem ${\bar{a}}_g$ we consider $R({\bar{a}}_g)$. The set of allocations given by this procedure is exactly

$$\begin{aligned} \bigcup _{{\bar{y}} \in LP(b(a))} \bigotimes _{g=1}^{h} R({\bar{a}}_g), \end{aligned}$$

where $\bigotimes$ denotes the Cartesian product.

This procedure provides a rule ${\bar{R}}$ which satisfies non-wastefulness, weak equal treatment of equals and non-manipulability by merging or splitting. Indeed, by definition and taking into account that *R* satisfies non-wastefulness and weak equal treatment of equals, ${\bar{R}}$ satisfies non-wastefulness and weak equal treatment of equals too.

Let $a=(N,H,p,c,E), a'=(N',H,p,c',E) \in {\mathbb {A}}$ with $N' \subset N$ such that for $i \in N'$ the following holds

1. $$c'_{ig} = c_{ig} + \sum _{j \in N \backslash N'} c_{jg}$$ for all $$g \in H$$
2. $c'_{jg}=c_{jg}$ for all $j \in N' \backslash \{i\}$ and for all $g \in H$

First of all, note that $LP(b(a))=LP(b(a'))$. Now let $x' \in {\bar{R}}(a')$, then there is an optimal solution ${\bar{y}}$ such that $x'_{g} \in R({\bar{a}}'_g)$ for all $g \in H$. Since $LP(b(a))=LP(b(a'))$ and *R* satisfies non-manipulability by merging or splitting, then for each $g \in H$ there is an $x_{g} \in R({\bar{a}}_g)$ such that $x'_{ig} = x_{ig} + \sum _{j \in N \backslash N'} x_{jg}$. The proof of the second condition is analogous. Therefore, ${\bar{R}}$ satisfies non-manipulability by merging or splitting. $\square$

The previous result states that, if we are able to obtain rules that satisfy the three conditions in a reduced domain of problems (with just one item), then they can be extended to the general domain. And conversely, if the three properties are not compatible when $|H|=1$, then they are not compatible in general. Theorem 2 exploits this relation to conclude that, in this setting, it is not possible to find a rule that fulfills non-wastefulness, weak equal treatment of equals and non-manipulability by merging or splitting.

### Theorem 2

*There is no rule that satisfies non-wastefulness, weak equal treatment of equals and non-manipulability by merging or splitting.*

### Proof

Let us consider $a=\left( N=\{1,2,3,4,5,6\}, H=\{1\}, p=(1), c=\left( 1,1,1,1,1,5\right) , E =4\right)$, and a rule *R* that satisfies non-wastefulness, non-manipulability by merging or splitting, and weak equal treatment of equals. Let us consider $a'=\left( N'=\{1,6\}, H=\{1\}, p=(1), c'=(5,5), E=4 \right)$, in this case by non-wastefulness and weak equal treatment of equals $R(a') =\{(2,2)\}$.

Now, let us consider

$$\begin{aligned}&a''=\left( N=\{1,2,3,4,5,6,7,8,9,10\}, H=\{1\}, p=(1), \right. \\&\quad \left. c=\left( 1,1,1,1,1,1,1,1,1,1\right) , E =4\right) , \end{aligned}$$

in this case, by non-wastefulness and weak equal treatment of equals $R(a'')$ is the set of all possible 0-1 vectors, such that the sum of their coordinates is 4. Therefore, by non-manipulability by splitting the allocation (0, 0, 0, 0, 0, 4) belongs to *R*(*a*). Again, by non-manipulability by splitting $R(a')=\{(2,2)\}$ is the unique result and the impossibility of $x_{1}^{\prime }=0$ is more evident. $\square$

Theorem 2 provides a surprising result, since it states an incompatibility among some principles that are compatible in the classical claims problem (see Thomson (2019)). Notice that none of the properties in the previous result is very demanding by itself. Indeed, the next propositions show that any pairwise combination of non-wastefulness, weak equal treatment of equals and non-manipulability by merging or splitting is feasible. Besides, the set of rules that satisfy each pairwise combination of properties is so wide that it does not seem to have a clear structure.

### Proposition 1

*There are rules that satisfy non-wastefulness and weak equal treatment of equals together.*

### Proof

To prove the result is sufficient to show that there is at least one rule satisfying these two properties for each $a \in {\mathbb {A}}$. We define the following rule $WE(a) = ILP(a) \cap E(a)$ for each $a \in {\mathbb {A}}$, where *E*(*a*) is the set of all weak equal treatment of equals allocations in *X*(*a*). Now we only need to prove that *WE*(*a*) is always nonempty for each $a \in {\mathbb {A}}$.

Let $a=(N,H,p,c,E) \in {\mathbb {A}}$ and let $i,j \in N$ be such that $c_{ig}=c_{jg}$ for all $g \in H$. Given an allocation $x^{*} \in ILP(a)$, let us suppose that there is some item $g \in H$, such that $|x_{ig}^{*}-x_{jg}^{*}|>1$. Now we consider the following allocation:

$$\begin{aligned} x'_{kf}={\left\{ \begin{array}{ll} x_{kf}^{*} &{} \text {if } k \ne i,j, \\ x_{kf}^{*} &{} \text {if } f \ne g, \\ min\{x_{ig}^{*},x_{jg}^{*}\} + \left\lfloor \frac{|x_{ig}^{*}-x_{jg}^{*}|}{2} \right\rfloor &{} \text {if } k=i \text { and } f =g, \\ min\{x_{ig}^{*},x_{jg}^{*}\} + \left\lfloor \frac{|x_{ig}^{*}-x_{jg}^{*}|}{2} \right\rfloor + \left\lceil m\{\frac{|x_{ig}^{*}-x_{jg}^{*}|}{2}\} \right\rceil &{} \text {if } k=j \text { and } f =g, \end{array}\right. } \end{aligned}$$

where given $r \in {\mathbb {R}}$, $\lfloor r \rfloor$ is the integer part of *r*, $\lceil r \rceil$ is the lowest integer larger than or equal to *r*, and $m\{r\}$ is the fractional part of *r*.

Finally, it is easy to check that $x' \in ILP(a)$ and, obviously, also $x' \in E(a)$, therefore $x' \in WE(a)$. Now, since $ILP(a) \ne \varnothing$ and $E(a) \ne \varnothing$ for each $a \in {\mathbb {A}}$, $WE(a) \ne \varnothing$ for each $a \in {\mathbb {A}}$. $\square$

### Proposition 2

*There are rules that satisfy non-wastefulness and non-manipulability by merging or splitting together.*

### Proof

To prove the result is sufficient to show that for each $a \in {\mathbb {A}}$, the set *ILP*(*a*) satisfies non-manipulability by merging or splitting.

Let $a=(N,H,p,c,E), a'=(N',H,p,c',E) \in {\mathbb {A}}$ with $N' \subset N$, and let $i \in N'$ such that the following two conditions hold

1. $$c'_{ig} = c_{ig} + \sum _{j \in N \backslash N'} c_{jg}$$ for all $$g \in H$$
2. $c'_{jg}=c_{jg}$ for all $g \in H$ and for all $j \in N' \backslash \{i\}$.

Let $x' \in ILP(a')$, then we define for each $j \in N$ and for each $g \in H$, the following allocation:

$$\begin{aligned} x_{jg}= {\left\{ \begin{array}{ll} x'_{jg} &{} \text {if } j \in N' \text { and } j \ne i, \\ GR((N \backslash N') \cup i, \left( c_{kg} \right) _{k \in (N \backslash N') \cup i},x'_{ig}) &{} \text {if } j \in (N \backslash N') \cup i, \end{array}\right. } \end{aligned}$$

where $GR((N \backslash N') \cup i, \left( c_{kg} \right) _{k \in (N \backslash N') \cup i},x'_{ig})$ is the application of the greatest remainder method^10^ to distribute $x'_{ig}$ according to the vector $\left( c_{kg}\right) _{k \in (N \backslash N') \cup i}$. Now, it is easy to check that this allocation *x* belongs to *ILP*(*a*).

Conversely, let $x \in ILP(a)$, then we define for each $j \in N'$ and for each $g \in H$, the following allocation:

$$\begin{aligned} x'_{jg}= {\left\{ \begin{array}{ll} x_{jg} &{} \text {if } j \in N' \text { and } j \ne i, \\ x_{ig} + \sum _{k \in N\backslash N'} x_{kg} &{} \text {if } j = i. \end{array}\right. } \end{aligned}$$

Again, it is easy to check that this allocation $x'$ belongs to $ILP(a')$. Therefore, the rule $R^{ILP}(a) = ILP(a)$ for each $a \in {\mathbb {A}}$ satisfies non-wastefulness and non-manipulability by merging or splitting. $\square$

### Proposition 3

*There are rules that satisfy weak equal treatment of equals and non-manipulability by merging or splitting together.*

### Proof

The null rule satisfies both non-manipulability by merging or splitting and weak equal treatment of equals. $\square$

## Protective Properties and Duality

In this section we study the compatibility between the non-wastefulness condition and other standard properties required when solving claims problems. In particular, we focus on requirements that protect small claimants. In some cases these properties establish the conditions under which an agent has such a small claim that she should be excluded from rationing. In other cases they guarantee a minimum amount of resources to each individual.

Consider an agent *i*, and replace any other agent’s claim (for all the items) by the claim of agent *i*. Imagine that in the new problem resulting from this replacement the overall demand does not exceed the available estate. *Exemption* states that, in such a case, the claim of *i* is so small that she is not responsible for the shortage, and she should be excluded from rationing. This is, *i* should receive her claim.^11^

**Exemption** For each $a \in {\mathbb {A}}$ and each $i \in N$, if

$$\begin{aligned} n \cdot \left( \sum _{g=1}^h p_g c_{ig} \right) \le E, \end{aligned}$$

then, for any $x \in R(a)$, $x_{ig} = c_{ig}$ $\forall g \in H$.

The next property applies a different criterion to determine when an agent has a small claim. Consider an agent *i*, and replace any other agent’s claim (for all the items) by the minimum between her claim and the claim of agent *i*. Imagine that in the new problem resulting from this replacement the overall demand does not exceed the available estate. *Conditional full compensation* states that, in such a case, agent *i* should be excluded from rationing and receive her whole claim.^12^

**Conditional full compensation** For each $a \in {\mathbb {A}}$ and each $i \in N$, if

$$\begin{aligned} \sum _{j \in N_i^{-}}\sum _{g=1}^h p_g c_{jg} + (n-|N_i^{-}|) \sum _{g=1}^h p_g c_{ig}\le E, \end{aligned}$$

then, for any $x \in R(a)$, $x_{ig} = c_{ig}$ $\forall g \in H$, where $N_i^{-} =\left\{ j \in N : \sum _{g=1}^h p_g c_{jg} < \sum _{g=1}^h p_g c_{ig}\right\}$.

Notice that exemption implies conditional full compensation, and both properties coincide when $|N|=2$.

### Theorem 3

*There is no rule that satisfies non-wastefulness and conditional full compensation together.*

### Proof

Let *R* be a rule that satisfies both properties in the statement of the theorem. Let us consider the problem where $N=\{1,2,3\}$, $H=\{1,2\}$, $p=(3,7)$, $E=35$, and

$$\begin{aligned} c= \left( \begin{array}{cc} 2 &{} 3 \\ 1 &{} 2 \\ 1 &{} 1 \end{array} \right) . \end{aligned}$$

For Claimant 3 we have that $N^-_3=\phi$ and therefore $\sum _{j \in N_3^{-}}\sum _{g=1}^2 p_g c_{jg} + (3-|N_3^{-}|) \sum _{g=1}^2 p_g c_{3g}=30 \le 35$. Since the rule satisfies *conditional full compensation*, it must happen that $x_{31} = x_{32}=1$, which is not compatible with non-wastefulness. Indeed, if $x_{31} = x_{32}=1$ then 25 units of estate remains. But this remaining estate cannot be allocated to Agents 1 and 2 fulfilling non-wastefulness, because the unique positive and integer linear combination of the numbers $p_1=3$ and $p_2=7$ is $6p_1 + 1p_2=25$. However, this would imply to assign 6 units of the first item, which exceeds the joint claim of Agents 1 and 2. $\square$

As a consequence of the previous result, neither exemption and non-wastefulness are compatible. Theorem 3 illustrates that, for the problem of adjudicating conflicting indivisible claims with different prices, efficiency (non-wastefulness) and some protective conditions (exemption or conditional full compensation) cannot be conciliated. It is worth noting that this impossibility is a particularity of the model with several items and prices. Both when claims and estate are divisible, and when they are expressed in indivisible units these two properties are compatible ( Herrero and Martínez (2008a)).

The next property, called *securement*, was introduced by Moreno-Ternero and Villar (2004) and guarantees a minimal share to every agent. More precisely, it imposes two conditions. One, an individual holding a feasible claim (the value of her demand at the prices of the items is below the estate) should receive allocations whose value is, at least, one nth of the value of her claim. And two, an individual holding an unfeasible claim (the value of her demand at the prices of the items is above the estate) should receive allocations whose value is, at least, one nth of the estate.

**Securement** For each $a \in {\mathbb {A}}$, each $x \in R(a)$, and each $i \in N$

$$\begin{aligned} \sum _{g=1}^h p_g x_{ig} \ge \frac{1}{n} \min \left\{ \sum _{g=1}^h p_g c_{ig}, E \right\} , \quad \forall i \in N. \end{aligned}$$

### Example 3

Let us consider the problem where $N=\{1,2,3\}$, $H=\{1,2\}$, $p=(2,4)$, $E=10$, and

$$\begin{aligned} c= \left( \begin{array}{cc} 1 &{} 2 \\ 1 &{} 2 \\ 1 &{} 2 \end{array} \right) \end{aligned}$$

For any agent $i \in N$, securement implies that $2x_{i1}+4x_{i2} \ge \frac{10}{3}$. Besides, by the definition of a rule, it must happen that $2(x_{11}+x_{21}+x_{31}) + 4(x_{12}+x_{22}+x_{32}) \le 10$ with $x_{ig} \in {\mathbb {Z}}_+$, but this is impossible.

The previous example illustrates that securement, as it is defined above, cannot be directly applied to this model because it is incompatible with the definition of a rule itself. The main reason is that the lower bound securement imposed is too high. Therefore, it must be definitively discarded.

In the spirit of securement, the next criterion guarantees to each agent a minimum amount that, at the same time, is also compatible with the existence of feasible allocations. To this end, we look for the largest lower bound of the value of the allocation of any agent $i\in N$. That is, we are looking for a value $\alpha _i$ such that (i) we can impose that $\sum _{g=1}^h p_g x_{ig} \ge \alpha _i$ (in the line of securement), (ii) $\alpha _i$ is compatible with the existence of feasible allocations, and (iii) if $\alpha _i$ increases by an infinitely small amount $\varepsilon \in {\mathbb {R}}_{++}$ then the impossibility emerges again.

**Weak securement** For each $a \in {\mathbb {A}}$, each $x \in R(a)$, and each $i \in N$

$$\begin{aligned} \sum _{g=1}^h p_g x_{ig} \ge \max _{y \in X(a)} \left\{ \sum _{g=1}^h p_g y_{ig} \left| \sum _{g=1}^h p_g y_{ig} \le \frac{1}{n} \min \left\{ \sum _{g=1}^h p_g c_{ig}, E \right\} \right. \right\} , \quad \forall i \in N. \end{aligned}$$

This is the explanation in detail of the previous alternative definition of securement. We know, by Example 3, that it must happen that $\sum _{g=1}^h p_g y_{ig} \le \frac{1}{n} \min \left\{ \sum _{g=1}^h p_g c_{ig}, E \right\}$ because otherwise we have impossibility. So, among those allocations that generate feasibility, we take those in which the claimant obtains the largest possible value. Thus, weak securement can be applied to this model without the issues originated by the standard definition of securement.

The next result shows that, however, when non-wastefulness is required in conjunction with weak securement, an impossibility emerges.

### Theorem 4

*There is no rule that satisfies non-wastefulness and weak securement together.*

### Proof

Let *R* be a rule that satisfies both properties in the statement of the theorem. Let us consider the problem where $N=\{1,2,3\}$, $H=\{1,2\}$, $p=(3,7)$, $E=14$, and

$$\begin{aligned} c= \left( \begin{array}{cc} 1 &{} 4 \\ 2 &{} 3 \\ 6 &{} 0 \end{array} \right) . \end{aligned}$$

Since *R* satifies *non-wastefulness* we have that

$$\begin{aligned} R(a) \subseteq \left\{ \left( \begin{array}{cc} 0 &{} 1 \\ 0 &{} 1 \\ 0 &{} 0 \end{array} \right) , \left( \begin{array}{cc} 0 &{} 2 \\ 0 &{} 0 \\ 0 &{} 0 \end{array} \right) , \left( \begin{array}{cc} 0 &{} 0 \\ 0 &{} 2 \\ 0 &{} 0 \end{array} \right) \right\} . \end{aligned}$$

Notice that, in any allocation $x \in R(a)$ the third agent does not receive any unit of any item. And hence, $3 x_{31} + 7 x_{32}=0$. This implies, because of *weak securement*, that $3 y_{31} + 7 y_{32}=0$ for all $y \in X(a)$ such that $3 y_{31} + 7 y_{32} \le \frac{1}{3}\min \{3 \cdot 6 + 7 \cdot 0, 14\}=\frac{14}{3}$. Which is not true. $\square$

The last of the criteria, called *self-duality*, was formulated by Aumann and Maschler (1985). It states that the problem of dividing profits should be solved symmetrically to the problem of dividing losses. Before defining the property we introduce the dual problem of a claim problem. Given $a = (c,E) \in {\mathbb {A}}$, the *associated dual problem* of *a* is given by $a^d =(c,L) \in {\mathbb {A}}$, where $L=\Vert c \cdot p\Vert - E$.

**Self-duality** For each $a \in {\mathbb {A}}$ it holds that $R(a)=c-R(a^d)$.

### Proposition 4

*If a rule satisfies the self-duality property then it exhausts the estate.*

### Proof

Let *R* be a rule that is self-dual but does not exhaust the estate. Then, for a given problem $a \in {\mathbb {A}}$ there exists an allocation $x \in R(a)$ such that

$$\begin{aligned} \sum _{i=1}^n \sum _{g=1}^h p_g x_{ig} < E \end{aligned}$$

In application of *self-duality*, $x^d = c - x \in R(a^d)$. However,

$$\begin{aligned} \sum _{i=1}^n \sum _{g=1}^h p_g x^d_{ig} = \sum _{i=1}^n \sum _{g=1}^h p_g c_{ig} - \sum _{i=1}^n \sum _{g=1}^h p_g x_{ig} > \Vert cp\Vert - E = L \end{aligned}$$

In other words, since $\Vert x^d \cdot p\Vert >L$, the allocation $x^d$ is not feasible and hence we have a contradiction. Therefore, if *R* is self-dual then the estate must be completely used. $\square$

The converse of Proposition 4 is not true in general. For example, if we consider the class of problems with $H=\{1\}$ and $p_1 = 1$ and *E* a positive integer number, then the discrete constrained equal awards rule (see, for example, Herrero and Martínez (2008a)) always exhaust the estate but does not satisfies self-duality.

An immediate consequence of Proposition 4 is that there can be no rules that satisfy the property of self-duality, since no rule can always exhaust the estate, in general. Notice that, unlike the other results in this section, the lack of self-dual rules is absolute, and the principle of non-wastefulness plays no role in that.

## An Alternative to Non-Wastefulness: Pareto Efficiency

As we have already mentioned, most of the models on claims problems assume that the estate must be fully distributed and nothing must remain. This requirement is called *balance* by Thomson (2019), but it is also known as *efficiency* (Thomson (2003), de Mesnard (2015)). It is quite obvious that this principle cannot be directly applied to our setting. In the previous sections we have interpreted this requirement from the point of view of a central authority whose goal is to do the most with the least, focusing on the use of the budget and trying to minimize the wasted estate. However, we have found this requirement to be very demanding and not compatible with many reasonable properties.

In this section we explore an alternative formulation of the efficiency principle: *Pareto efficiency*. In contrast with non-wastefulness, this property focuses on the agents’ allocations rather than on the expenditure of the budget. An allocation is Pareto efficient if there is no other allocation in which some individual is better off and no individual is worse off.

### Definition 1

For $a \in {\mathbb {A}}$, $x \in X(a)$ is *Pareto efficient* if there is no other allocation $x' \in X(a)$ such that $\sum _{g \in H}p_gx'_{ig} \ge \sum _{g \in H}p_gx_{ig}, \forall i \in N$, with at least one strict inequality.

Given $a \in {\mathbb {A}}$, we denote by $P(a)\subset X(a)$ the set of all allocations which are Pareto efficient.

**Pareto efficiency** For $a \in {\mathbb {A}}$, $R(a) \subseteq P(a)$.

Notice that it is glaringly obvious that non-wastefulness implies Pareto efficiency, but the converse is not true. Even though these two properties are not equivalent in general, it is not difficult to prove that they coincide when $|H|=1$. As a consequence, we can replace non-wastefulness by Pareto efficiency in Theorem 1, which implies that weak equal treatment of equals and non-manipulability by merging or splitting together are incompatible with Pareto efficiency. This result is the analogous to Theorem 2.

### Theorem 5

*There is no rule that satisfies Pareto efficiency, weak equal treatment of equals and non-manipulability by merging or splitting.*

Since Pareto efficiency is milder than non-wastefulness, we obtain the counterparts of Propositions 1 and 2.

### Proposition 5

*There are rules that satisfy Pareto efficiency and weak equal treatment of equals together.*

### Proposition 6

*There are rules that satisfy Pareto efficiency and non-manipulability by merging or splitting together.*

With regard to self-duality, it is evident that it will not be compatible with Pareto efficiency, since the latter does not guarantee that the estate is fully exhausted. Theorems 3 and 4 state that conditional full compensation and weak securement are incompatible with non-wastefulness. However, the next two results show that, if the latter requirement is weakened to Pareto efficiency, then the possibility emerges.

### Theorem 6

*There are rules that satisfy Pareto efficiency and conditional full compensation together.*

### Proof

In order to prove this result is sufficient to show that there is at least one rule satisfying both properties. Given a problem $a=\left( N,H,p,c,E\right)$, we proceed as follows:

- The agents are ordered according to their claims on the budget in this way,
  $$\begin{aligned} i \preceq j \Leftrightarrow \sum _{g=1}^{h}p_gc_{ig}\le \sum _{g=1}^{h}p_gc_{jg} \end{aligned}$$
  For each $i \in N$, we denote by $N_i^{-} =\left\{ j \in N : \sum _{g=1}^h p_g c_{jg} < \sum _{g=1}^h p_g c_{ig}\right\}$.
- Let $i_0$ be an agent in the previous order such that the following inequalities hold
  $$\begin{aligned} \sum _{j \in N_i^{-}}\sum _{g=1}^h p_g c_{jg} + (n-|N_{i_{0}}^{-}|) \sum _{g=1}^h p_g c_{i_{0}g}\le E, \end{aligned}$$
  and, for each $k \in N$ such that $i_{0} \in N_{k}^{-}$,
  $$\begin{aligned} \sum _{j \in N_k^{-}}\sum _{g=1}^h p_g c_{jg} + (n-|N_{k}^{-}|) \sum _{g=1}^h p_g c_{kg} >E. \end{aligned}$$
  Moreover, we denote by $N^0=\left\{ i \in N: i\preceq i_0\right\}$. Note that $N^0$ is independent of the chosen agent $i_0$.
- Let $x^0$ be the following allocation, $x^0_{ig}=c_{ig}, \forall g\in H$, if $i \in N^0$, and $x^0_{ig}=0, \forall g\in H$, otherwise.

We define $X^0(a) = \{x \in X(a)\left| x - x^0 \in X(a) \right. \}$. Now, we define the following rule

$$\begin{aligned} R^C(a) =x^0+ILP(a'), \end{aligned}$$

where $$a'=\left( N,H,p,c-x^0,E-\Vert x^0\cdot p\Vert \right) $$.

By definition this rule satisfies Pareto efficiency and conditional full compensation. $\square$

### Theorem 7

*There are rules that satisfy Pareto efficiency and weak securement together.*

### Proof

Again, to prove this result is sufficient to show that there is at least one rule satisfying both properties. Given a problem $a=\left( N,H,p,c,E\right)$, we proceed as follows:

First, we consider the set $X^{WS}(a) \subset X(a)$ given by

$$\begin{aligned} x \in X^{WS}(a) \;\Leftrightarrow & {} \; \sum _{g=1}^h p_g x_{ig} \ge \max _{y \in X(a)} \left\{ \sum _{g=1}^h p_g y_{ig} \left| \sum _{g=1}^h p_g y_{ig} \le \frac{1}{n}\min \left\{ \sum _{g=1}^h p_g c_{ig}, E \right\} \right. \right\} ,\\&\qquad \forall i \in N. \end{aligned}$$

Now, we define the following rule

$$\begin{aligned} R^{S}(a)= X^{WS}(a) \cap P(a). \end{aligned}$$

By definition $R^{S}$ satisfies weak securement and Pareto efficiency. Furthermore, this rule is nonempty. Indeed, consider $R^{EA}(a)$ that we know it is nonempty. If there is an allocation $x \in R^{EA}(a)$ that is Pareto efficient, then $x \in R^{S}(a)$. Otherwise, for each $x \in R^{EA}(a)$ there exists $x' \in X(a)$ such that $\sum _{g \in H}p_gx'_{ig} \ge \sum _{g \in H}p_gx_{ig}, \forall i \in N$, with at least one strict inequality, but this $x' \in X^{WS}(a)$. If $x'$ is not Pareto efficient, we can find another $x'' \in X(a)$ such that $\sum _{g \in H}p_gx''_{ig} \ge \sum _{g \in H}p_gx'_{ig}, \forall i \in N$, with at least one strict inequality. Furthermore,

$$\begin{aligned} \sum _{i \in N} \sum _{g \in H}p_gx_{ig}< \sum _{i \in N} \sum _{g \in H}p_gx'_{ig} < \sum _{i \in N} \sum _{g \in H}p_gx''_{ig} \le E. \end{aligned}$$

Now, since *X*(*a*) is a finite set there exists $K > 0$ such that any positive improvement of an agent from one allocation to another is larger than or equal to *K*, i.e., *K* is the minimal positive improvement that an agent can obtain from one allocation to another. Therefore, since *E* is finite the above chain of allocations cannot be continued indefinitely, so that there will be an allocation in $X^{WS}(a)$ that it is Pareto efficient. $\square$

Therefore, Pareto efficiency is a sufficiently less demanding property to be compatible with other reasonable properties. Furthermore, we can define rules that satisfy several of the properties introduced in this paper. For example, the following rule

$$\begin{aligned} R^{CS}(a)=x^0 + R^{S}(a'), \forall a \in {\mathbb {A}}, \end{aligned}$$

where $a'=\left( N,H,p,c-x^0,E-\Vert x^0\cdot p\Vert \right)$, satisfies Pareto efficiency, conditional full compensation, and weak securement.

Consider the rule $R^{CES}$ defined as follows. For each, $a \in {\mathbb {A}}$,

$$\begin{aligned} R^{CES}(a)=R^{CS}(a) \cap E(a) \end{aligned}$$

This rule satisfies Pareto efficiency, weak equal treatment of equals, conditional full compensation, and weak securement. The converse is not true, there are rules different from $R^{CES}$ that also fulfill these four properties. However, any rule that satisfies Pareto efficiency, weak equal treatment of equals, conditional full compensation, and weak securement must be a subselection of $R^{CES}$.

### Theorem 8

*If a rule* *R* *satisfies Pareto efficiency, weak equal treatment of equals, conditional full compensation, and weak securement, then* $R(a) \subseteq R^{CES}(a)$, $\forall a \in {\mathbb {A}}$.

### Proof

Let *R* be a rule satisfying Pareto efficiency, equal treatment of equals, conditional full compensation, and weak securement. Let $a \in {\mathbb {A}}$ and $x \in R(a)$. Since *R* satisfies conditional full compensation, *x* can be written as $x^0 + (x - x^0)$ so that $(x - x^0) \in X^0(a)$.

Since *R* satisfies weak securement, we have that for each *i* such that $x_{ig}^0 =0, \forall g \in H$,

$$\begin{aligned} \sum _{g=1}^h p_g x_{ig} \ge \max _{y \in X(a)} \left\{ \sum _{g=1}^h p_g y_{ig} \left| \sum _{g=1}^h p_g y_{ig} \le \min \left\{ \frac{\sum _{g=1}^h p_g c_{ig}}{n},\frac{E}{n}\right\} \right. \right\} , \end{aligned}$$

otherwise, by conditional full compensation $x_{ig}^0 =c_{ig}, \forall g \in H$. Moreover, *R* satisfies Pareto efficiency. Therefore, $x \in R^{CS}(a)$.

Finally, since *R* satisfies weak equal treatment of equals, $x \in E(a)$. Therefore, $x \in R^{CS}(a) \cap E(a) = R^{CES}(a)$. $\square$

We finish with a table that summarizes the properties each rule in Sect. 2 satisfies (Table 1).

**Table 1:** Y means that the rule fulfills the property and N means that it does not

	Non-wastefulness	Pareto efficiency	weak equal treatment of equals	Non-manipulability	Exemption	Conditional full compensation	Weak securement	Self-duality
Null rule	N	N	Y	Y	N	N	N	N
Greedy rule	N	N	N	Y	Y	N	N	N
Agent-item priority arrival rule	N	N	N	.	N	N	N	N
Agent priority arrival rule	N	Y	N	.	N	N	N	N
Equal-by-item rule	N	N	Y	N	N	N	N	N
Equal-by-agent rule	N	N	Y	N	Y	Y	Y	N

We do not analyze the non-manipulability of the agent-item priority arrival and the agent priority arrival rules because it is unclear the effect on the ordering of merging or splitting agents

## Discussion

In this paper we have studied a particular class of claims problems. In our model a group of agents demand several units of different items, each of which has a price. The available estate is not sufficient to satisfy the aggregate claim. A rule is a multi-valued function that selects a set of allocations, which indicate the amount of units of each item that is assigned to each claimant.

In contrast with other models involving claims problems, efficiency cannot be guaranteed. The closest requirement is non-wastefulness, which states that the rule should waste as little estate as possible, and is closely related to the so-called bounded knapsack problem, whose solutions, in general, are difficult to obtain. Even though, with this milder condition of efficiency, we find that there is no rule that satisfies non-wastefulness together with other criteria that protect small agents or ensure claimants receive a minimum allocation.

In view of all the impossibility results obtained in this work, we can observe that it is not easy to reconcile efficiency (via non-wastefulness) with fairness. At the point we can follow to different paths. First, we contemplate an alternative notion of efficiency that weakens non-wastefulness. Or second, we reconsider the absolute necessity of the non-wastefulness property and simply guarantee that the maximum amount of estate is distributed, while respecting certain properties of fairness in the distribution. With regard to the first possibility, in Sect. 6 we analyze the implications of Pareto efficiency as a milder requirement of efficiency. Even though some impossibilities persist, we find out that Pareto efficiency is compatible with protective properties, in contrast with non-wastefulness. As for the second possibility, it is a promising research line which is beyond the objectives of this paper.

Finally, we should acknowledge there are several extensions of the model that are not addressed in this work. For example, Carpente et al. (2013) consider that, in addition to the claims, each agent is endowed with an utility function. In our model we obviate the latter element, which may be relevant in some situations. However, we do not expect that the addition of utilities as in Carpente et al. (2013) alters the main conclusions significantly, which rely on the discreteness of the claims (or, eventually, the utility of those claims) and on the multi-valued rules. For instance, with respect to non-wastefulness, the solution to the optimization problem in Equation (1) would take different values, but the structure (and implications) of the optimization program itself will still be the same. It is also worth mentioning that we do not provide any characterization in this paper. This is a very natural and convenient extension of this work, which we leave for further research. In contrast with other models in the literature, we define a rule as a correspondence, instead of a single-valued function. This change adds an extra layer of complexity both to the rules and to the axioms. When we try to extend the axioms from functions to correspondences many alternative arise. Think, for example, in properties that compares the outcome of two or more different problems: *additivity*, *composition*, *monotonicity*, *consistency*, etc. When rules are single-valued functions the comparison between two outcomes ($x=y$ or $x \le y$) is straightforward. When rules are multi-valued functions, as they are in our model, the comparison between two outcomes is not so evident ($R(a)=R(a')$, $R(a) \subset R(a')$, $R(a') \subset R(a)$, etc). That is, each axiom may have different and natural extensions. In this paper we have focused on the implications of one primary requirement: *efficiency*. Other principles and their consequences deserve a deeper analysis, which exceeds the purposes of this paper.

## Footnotes

1 Thomson (2003, 2015) are two excellent surveys on this literature.

2 We should point out that if we consider situations in which the claims are also perfectly divisible, the so-called multi-issue situations introduced by Calleja et al. (2005) arise.

3 Because of the particular nature of the kind of situations we are dealing with (continuous estate and discrete claims) we cannot impose that the rule must exhaust the estate completely. And this is a particularity of our model that differs from other works, since in the vast majority of the papers in the literature, both in continuous and discrete settings, the estate is completely allocated, and nothing remains.

4 Bounded knapsack problems are knapsack problems in which the variables are bounded from above.

5 This property states that if the claim of agent *i* is larger than the claim of agent *j*, she should obtain (lose) at least (at most) as much as agent *j*, and if two agents have equal claims, their amounts should differ at most by one unit.

6 For a given matrix *A* we denote by $\Vert A\Vert$ the norm 1 of *A*, that is, the sum of all the entries of the matrix.

7 The second step of this procedure is closely related to some other extensions in settings with indivisibilities of the so-called *constrained equal awards rule* (Herrero and Martínez 2008a, Chen 2015).

8 It was first studied in O’Neill (1982) with the name *strategy-proofness*, and analyzed in works as de Frutos (1999) or Ju et al. (2007).

9 Notice that the constrains $0 \le x_{ig} \le c_{ig}, \; \forall i \in N, \forall g \in H$ restrict the possible values of the optimization variables, and therefore the knapsack problem is bounded.

10 This is a well-known method in apportionment problems also known as the method of largest fractions or the Hare Quota method (see, for instance, Lucas (1982)). The integer budget is distributed proportionally to the integer claims, if the allocation is integer for all claimants that is the final allocation, if not, each claimant receives the integer part of his allocation, and an extra unit is allocated to the claimants with the highest fractional parts until the estate is exhausted.

11 This property was introduced by Herrero and Villar (2001).

12 This requirement is called *sustainability* in Herrero and Villar (2002).
